# Supplementary material for: Shade-induced RTFL/DVL peptides negatively regulate the shade response by directly interacting with BSKs in Arabidopsis
Source: Nat Commun. 2023 Oct 28;14:6898. doi: 10.1038/s41467-023-42618-3 (PMC10613268; doi:10.1038/s41467-023-42618-3)
Supplement: Supplementary file 1 — Supplementary Information [file 41467_2023_42618_MOESM1_ESM.pdf]

Supplementary Information

Shade-induced RTFL/DVL peptides negatively regulate the shade response by directly interacting with BSKs in Arabidopsis

Sha Huang[1], Yu Ma[1], Yitian Xu[1], Pengfei Lu[1], Jie Yang[2], Yu Xie[1], Jianhua Gan[2], Lin Li[1,*]

[1]State Key Laboratory of Genetic Engineering, Institute of Plants Biology, School of Life Sciences, Fudan University, Shanghai 200438, China
[2]Shanghai Public Health Clinical Center, State Key Laboratory of Genetic Engineering, Department of Physiology and Biophysics, School of Life Sciences, Fudan University, Shanghai 200438, China
[*]Corresponding Author Email: linli@fudan.edu.cn

The following Supplementary Information is available for this article:
Supplementary Fig. 1- Supplementary Fig.7
Supplementary Table 1

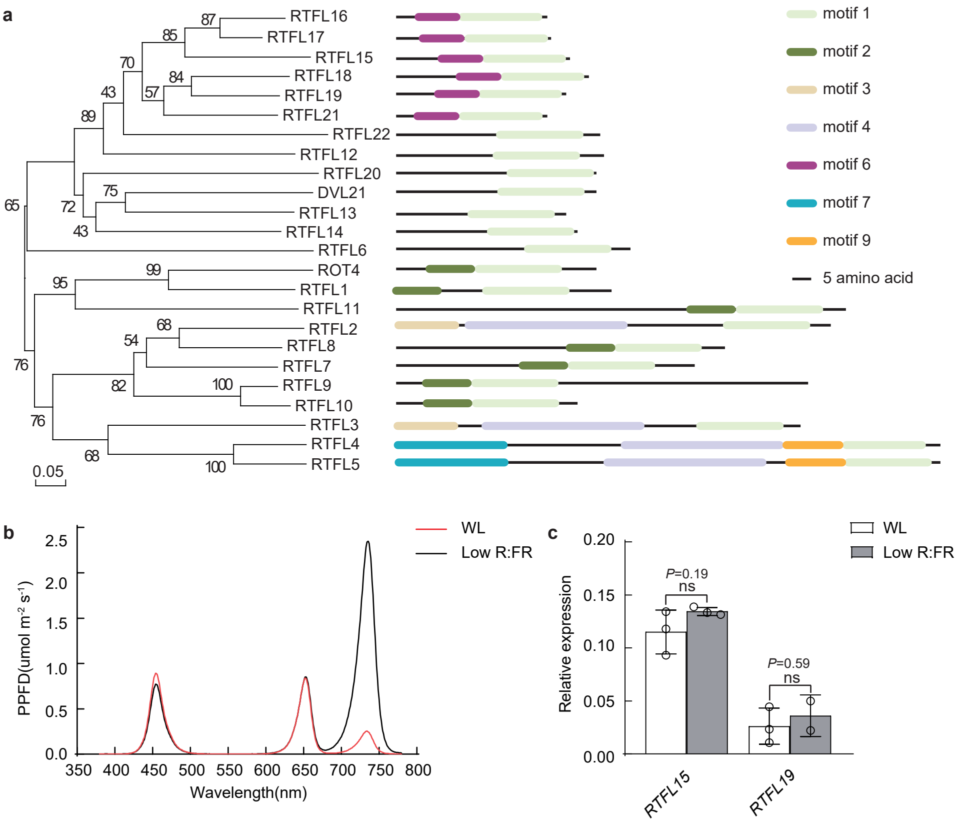

Supplementary Fig. 1 | Low R:FR light significantly regulates the transcription of at least five *RTFL/DVL* genes. a, Phylogenetic analysis of twenty-four *Arabidopsis* RTFL peptides. Evolutionary analysis was conducted in DNAMAN with a bootstrap of 1000. Motifs refer to those reported by Guo *et al*., 2015[1]. **b**, Light spectral composition of white light and low R:FR treatments used in this study. **c**, The relative expression levels of *RTFL15* and *RTFL19* in seedlings grown under WL and low R:FR conditions. Seedlings grown for 5 days under white light were transferred to low R:FR or continual white light exposure for 1 h. The expression levels of *RTFLs* were normalized against the expression of the reference gene *AT2G39960*. Data are presented as mean values +/- SD (n=3, n refers to biological replicates). The asterisks indicate significant differences to WL, respectively (Multiple *t* test: False Discovery Rate approach, ns indicates no significance). Source data are provided as a Source Data file.

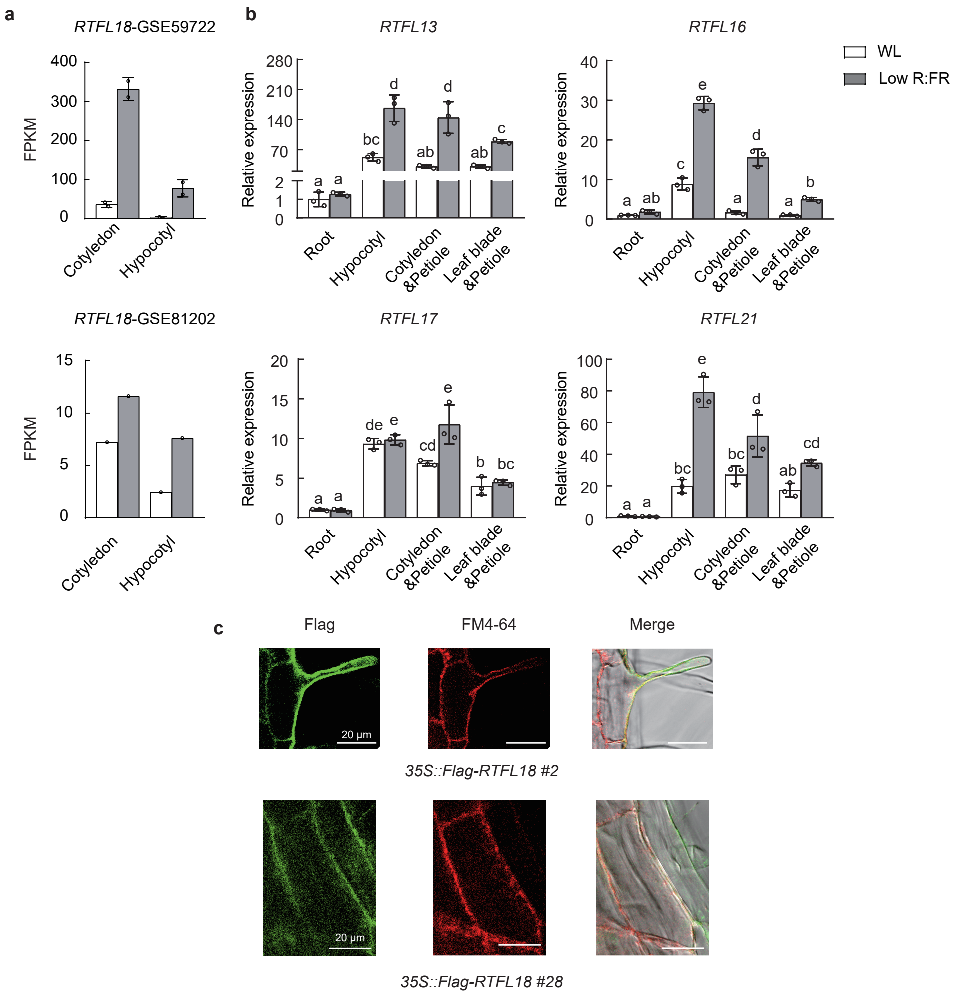

Supplementary Fig. 2 | Transcription levels of *RTFLs* in different tissue and subcellular localization of Flag-RTFL18. a, Transcription levels of *RTFL18* in cotyledons and hypocotyls in the GSE59722 and GSE81202 datasets. **b**, The relative expression of *RTFL13/16/17/21* in different tissues under WL and low R:FR conditions was measured by RT–qPCR. Seedlings grown for 10 days with white light were transferred to low R:FR conditions or continually exposed to white light for 1 h, and the different tissues were separated. The expression levels of *RTFL13/16/17/21* were normalized against the expression of the reference gene *AT2G39960*, and the expression in the roots under WL was standardized to be "1". Data are presented as mean values +/- SD (n=3, n refers to biological replicates). Letters indicate significant differences between mean values (one-way ANOVA: Tukey's multiple comparisons test, $P < 0.05$), and groups with the same letters are not significantly different. **c**, Subcellular localization of Flag-RTFL18 was measured by immunolocalization assay. Seedlings were grown under white light for 6 days. Flag-RTFL18 (green) was labeled by immunolocalization using anti-Flag antibodies and observed with a confocal laser scanning microscope. The membrane was stained with FM4-64 (red). Flag, Flag signal; FM4-64, FM4-64 staining; Merge, merged image of the Flag signal with the FM4-64 signal in a bright field. Scale bars represent 20 μm. Source data are provided as a Source Data file.

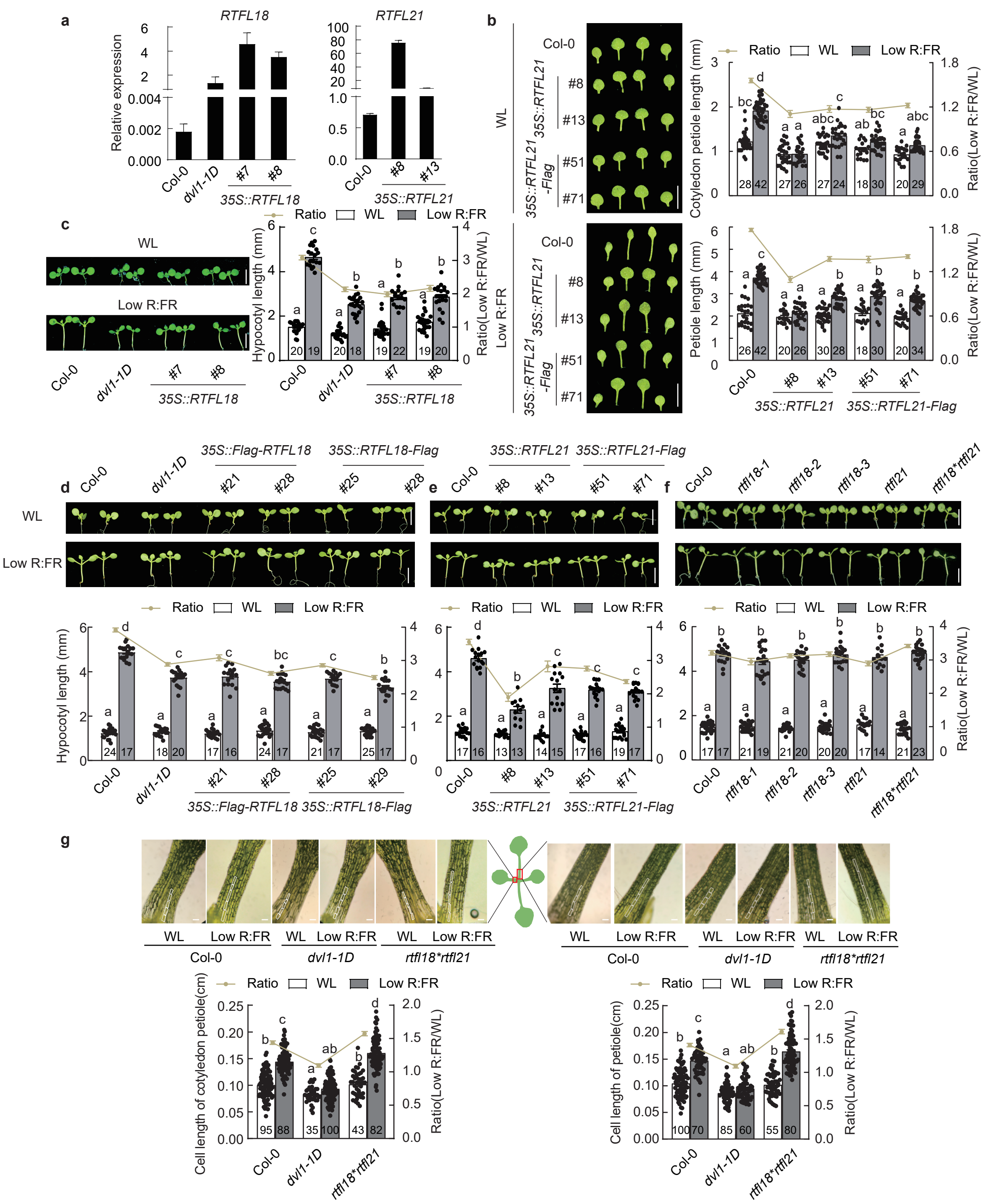

Supplementary Fig. 3 | Expression of *RTFL18* and phenotype of RTFL18-related mutants. a, The relative expressions of *RTFL18* or *RTFL21* in the Col-0, *dvl1-1D*, *35S::RTFL18* (*#7*, *#8*) lines and *35S::RTFL21* (*#8*, *#13*) lines were measured by RT–qPCR. Seedlings were grown under white light for 5 days. The expression levels of *RTFL18* were normalized against the expression of the reference gene *AT2G39960*. Data are presented as mean values +/- SD (n=3, n refers to biological replicates). **b**, The petiole lengths of the cotyledons and the first and second leaves in Col-0, *35S::RTFL21* (*#8, #13*), and *35S::RTFL18-Flag* (*#51, #71*) lines grown under white light (WL) and low R:FR conditions were measured. **c**, The hypocotyl lengths of the Col-0, *dvl1-1D,* and *35S::RTFL18* (*#7*, *#8*) lines grown under white light (WL) and low R:FR conditions. **d**, The hypocotyl lengths of the Col-0, *dvl1-1D*, *35S::Flag-RTFL18* (*#21*, *#28*), and *35S:: RTFL18-Flag* (*#25*, *#29*) lines grown under WL and low R:FR conditions. **e**, The hypocotyl lengths of the Col-0, *35S::RTFL21*, and *35S::RTFL18-Flag* lines grown under white light (WL) and low R:FR conditions. **f**, The hypocotyl lengths of the Col-0, *rtfl18-1*, *rtfl18-2*, *rtfl18-3*, *rtfl21*, and *rtfl18*rtfl21* lines grown under WL and low R:FR conditions. **g**, The cell lengths of the cotyledon petiole and the first/second petiole of the Col-0, *dvl1-1D*, and *rtfl18*rtfl21* lines grown under WL and low R:FR conditions. Seedlings grown for 3 days under white light were transferred to low R:FR conditions or continued to be grown under white light for 8 days.

The numbers in the bar charts represent n of each sample. Data are presented as mean values +/- SEM. Letters in **b**, **c**, **d**, **e**, **f**, and **g** indicate significant differences between mean values (two-way ANOVA: Tukey's multiple comparisons test, $P < 0.01$), and groups with the same letters are not significantly different. Scale bars represent 5 mm (**b**, **c**, **d**, **e**, **f**) and 100 μm (**g**). Source data are provided as a Source Data file.

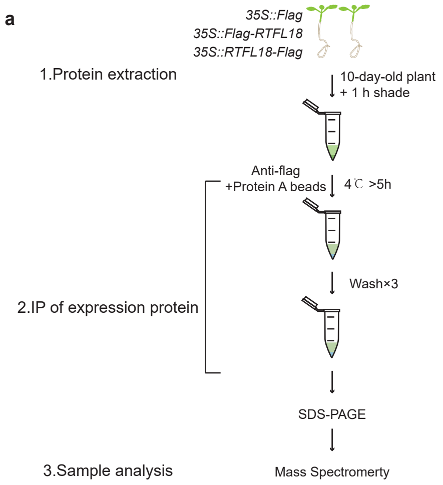

b

Accession	Coverage [%]	Unique Peptides	Description
AT1G63500.1	7	3	BR-signaling kinase 7(BSK7)
AT4G35230.1	4	1	BR-signaling kinase 1(BSK1)
AT1G13320.1	4	1	protein phosphatase 2A subunit A3(PP2AA3)
AT3G21220.1	3	1	MAP kinase kinase 5(MAPKK5)
AT4G01370.1	3	1	MAP kinase 4(MAPK4)
AT3G54030.1	2	1	BR-signaling kinase 6(BSK6)
AT2G43790.1	7	1	MAP kinase 6(MAPK6)
AT3G45640.1	7	1	mitogen-activated protein kinase 3(MAPK3)
AT5G03280.1	1	1	NRAMP metal ion transporter family protein(EIN2)
AT1G70940.1	6	3	Auxin efflux carrier family protein(PIN3)
AT3G02260.1	3	13	auxin transport protein (BIG)
AT4G23180.1	2	1	cysteine-rich RECEPTOR-like protein kinase 10(CRK10)

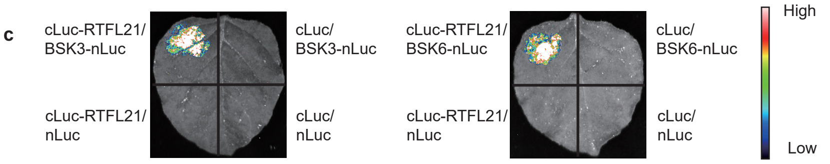

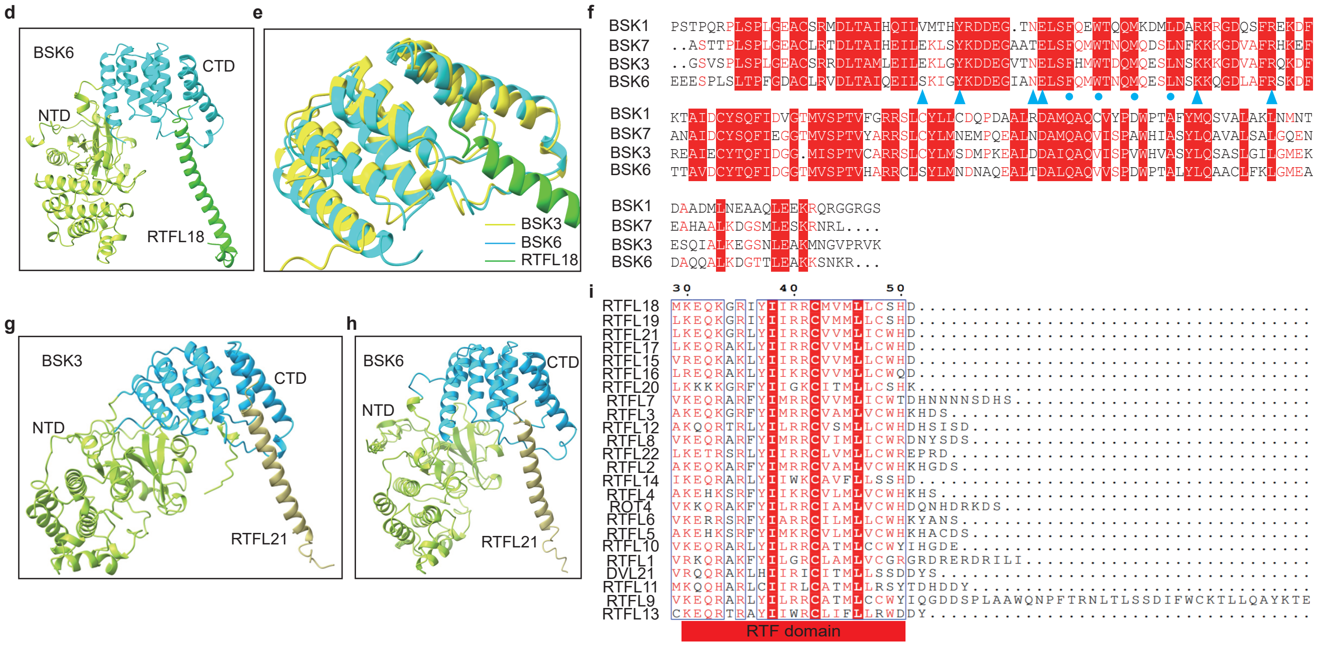

Supplementary Fig. 4 | RTFLs interact with BSK3/6. a, Schematic diagram of IP-MS. Seedlings grown for 10 days under white light were transferred to low R:FR and grown for 1 h. Total protein was extracted from *35S::Flag*, *35S::Flag-RTFL18,* and *35S::RTFL18-Flag* seedlings. **b**, Candidate proteins interacting with RTFLs as determined by MS. **c**, Interactions between RTFL21 and BSK3/BSK6 were detected by luciferase complementary imaging (LCI). The C-terminus of luciferase was fused to RTFL21, and the N-terminus of luciferase was fused to BSK3/BSK6. The constructs were cotransformed into *N. benthamiana* leaves. The luciferase signals were observed 60 h after infiltration. **d**, The BSK6/RTFL18 complex was predicted by AlphaFold-Multimer. The N-terminal domain (NTD) and C-terminal domain (CTD) of BSK6 are green yellow and cyan, respectively. RTFL18 is green. **e**, Superposition of the BSK3/RTFL18 and BSK6/RTFL18 complexes was predicted by AlphaFold-Multimer. The CTD domains of BSK3 and BSK6 are yellow and cyan, respectively. For clarity, only one RTFL18 is shown. **f**, Sequence alignment of the CTD domains of BSKs was conducted with GeneDoc. Conserved residues are highlighted. The residues involved in RTFL18 interactions are denoted with triangles (for H-bonds or electrostatic interactions) or dots (for hydrophobic interactions). **g,** The BSK3/RTFL21 complex was predicted by AlphaFold-Multimer. The N-terminal domain (NTD) and C-terminal domain (CTD) of BSK3 are green yellow and cyan, respectively. RTFL21 is yellow. **h**, The BSK6/RTFL21 complex was predicted by AlphaFold-Multimer. The N-terminal domain (NTD) and C-terminal domain (CTD) of BSK6 are green yellow and cyan, respectively. RTFL21 is yellow. **i**, Sequence alignment of the RTF domains in RTFL peptides was conducted with GeneD-oc. Conserved residues are highlighted. The RTF domain refers to that in a report by Guo *et al.*, 2015[1].

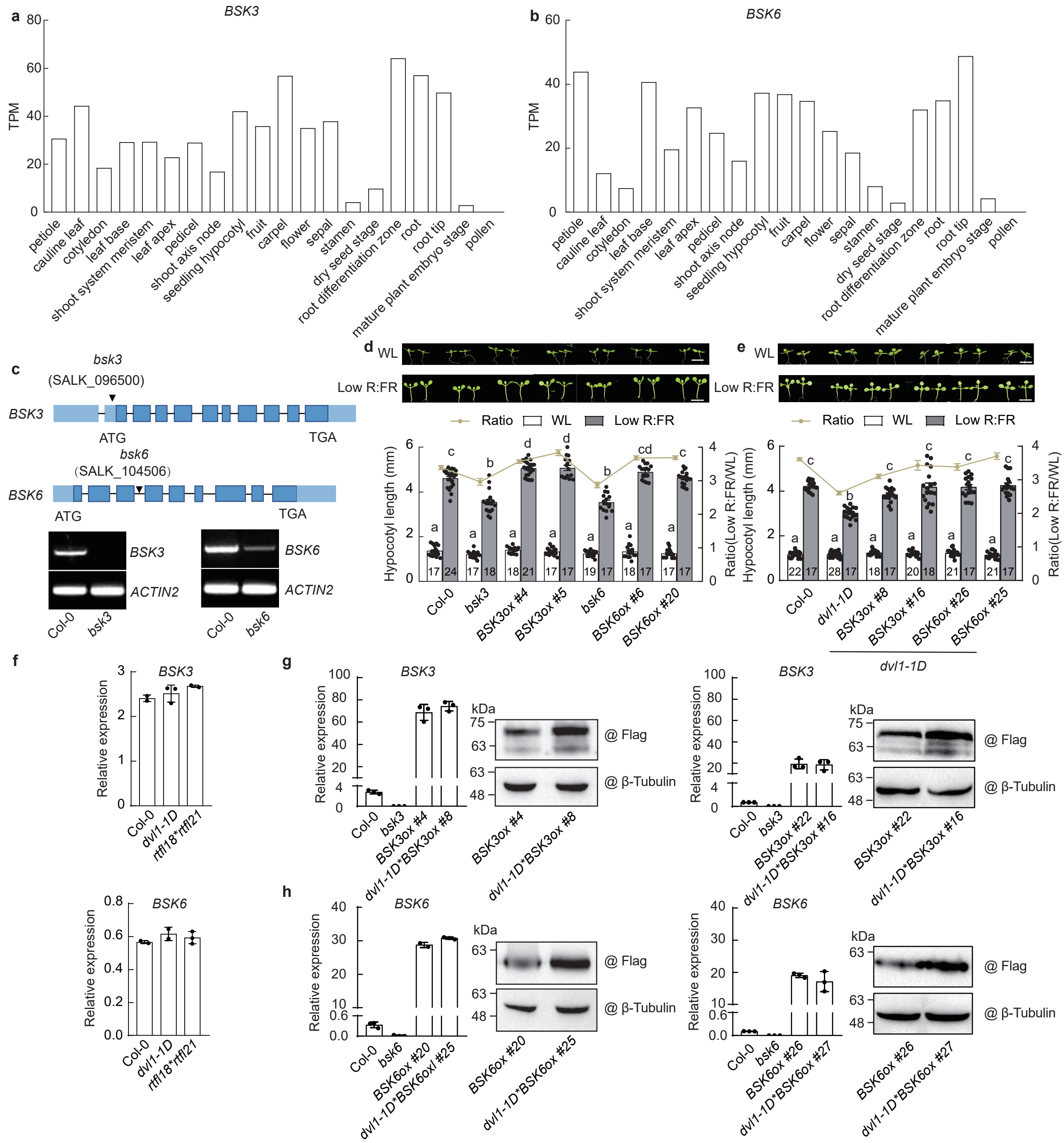

Supplementary Fig. 5 | RTFL18 represses BSK3/6-mediated BR signaling. a-b, The expressions of *BSK3* and *BSK6* in different tissues. Data were obtained from www.proteomicsdb.org. **c**, Schematic diagrams of *bsk3* and *bsk6* mutants. Light blue boxes represent the 5' or 3' UTR. Dark blue boxes represent exons. Lines represent introns. The expression levels of *BSK3* and *BSK6* in Col-0, *bsk3*, and *bsk6* lines were measured by RT–PCR. Seedlings were grown for 5 days under white light. *ACTIN2* was used as the internal control. **d**, The hypocotyl lengths of the Col-0, *bsk3*, *bsk6*, *BSK3ox*, and *BSK6ox* lines grown under WL and low R:FR conditions. **e**, The hypocotyl lengths of the Col-0, *dvl1-1D*, *dvl1-1D*BSK3ox*, and *dvl1-1D*BSK6ox* lines grown under WL and low R:FR conditions. **f,** The relative expression levels of *BSK3* and *BSK6* in the Col-0, *dvl1-1D*, and *rtfl18*rtfl21* lines were measured by RT–qPCR. **g-h,** The expression and protein levels of BSK3 and BSK6 in BSK-overexpressing seedlings were measured by RT–qPCR and western blotting. The left bar charts represent the relative expression levels of *BSK3/6* in Col-0, *bsk3*, *BSK3ox* and *dvl1-1D*BSK3ox*, or Col-0, *bsk6*, *BSK6ox* and *dvl1-1D*BSK6ox*. The right images represent the protein levels of *BSK3ox* and *dvl1-1D*BSK3ox*, or *BSK6ox* and *dvl1-1D*BSK6ox*.

In **d** and **e**, the numbers in the bar charts represent n of each sample. Data are presented as mean values +/- SEM. Letters indicate significant differences between mean values (two-way ANOVA: Tukey's multiple comparisons test, $P < 0.01$), and groups with the same letters are not significantly different. Scale bars represent 5 mm. In **f**, **g**, and **h**, seedlings were grown under white light for 5 days. The expression levels of *BSKs* were normalized against the expression of the reference gene *AT2G39960*. Data are presented as mean values +/- SD. In **g** and **h**, β-tubulin was used as the internal control and each experiment was repeated three times with similar results. Source data are provided as a Source Data file.

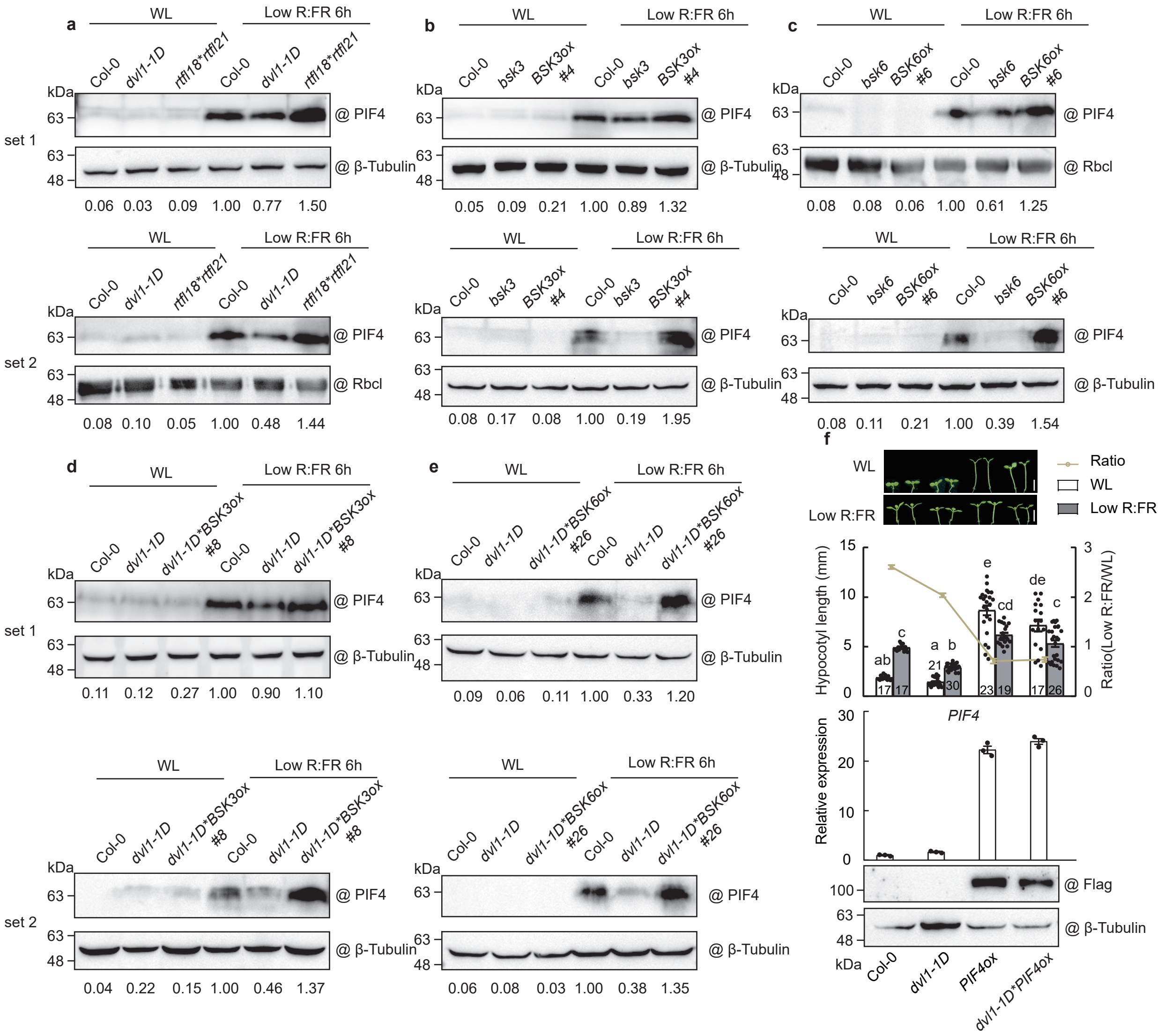

Supplementary Fig. 6 | RTFL18 antagonizes the stabilization of PIF4 under low R:FR conditions. **a**, The PIF4 protein levels in Col-0, *dvl1-1D*, and *rtfl18*rtfl21* lines grown under white light (WL) and low R:FR conditions. **b-c**, The PIF4 protein levels in the Col-0, *bsk3*, *BSK3ox #4*, *bsk6*, and *BSK6ox #6* lines under WL and low R:FR conditions. **d-e**, The PIF4 protein levels in the Col-0, *dvl1-1D*, *dvl1-1D*BSK3ox #8*, and *dvl1-1D*BSK6ox #26* lines under WL and low R:FR conditions. **f**, The hypocotyl lengths in the Col-0, *dvl1-1D*, *PIF4ox*, and *dvl1-1D*PIF4ox* lines grown under WL and low R:FR conditions. The numbers in the bar charts represent n of each sample. Data are presented as mean values +/- SEM. Letters indicate significant differences between mean values (two-way ANOVA: Tukey's multiple comparisons test, $P < 0.01$), and groups with the same letters are not significantly different. Scale bars represent 5 mm. The middle panel bar charts represent the relative expression levels of *PIF4* in Col-0, *dvl1-1D*, *PIF4ox*, and *dvl1-1D*PIF4ox*. Seedlings were grown under white light for 5 days. The expression levels of *PIF4* were normalized against the expression of the reference gene *AT2G39960*. The expression levels of *PIF4* in Col-0 were standardized as "1". Data are presented as mean values +/- SD. (n=3, n refers to biological replicates). The bottom panel represent the protein level of PIF4-Flag (*35S::PIF4-Flash,* 9×Myc-6×His-3×Flag) in Col-0, *dvl1-1D*, *PIF4ox*, and *dvl1-1D*PIF4ox*. Western blots were probed with an anti-Flag antibody. β-tubulin was used as the internal control.

In **a**, **b**, **c**, **d**, and **e**, the PIF4 protein levels were normalized to β-tubulin/ Rbcl. The PIF4 protein levels of Col-0 under low R:FR were set as 1. Western blots were probed with an anti-PIF4 antibody. The levels of β-tubulin/ Rbcl indicate the loading concentration. In **a-e**, each experiment was repeated three times with similar results. Source data are provided as a Source Data file.

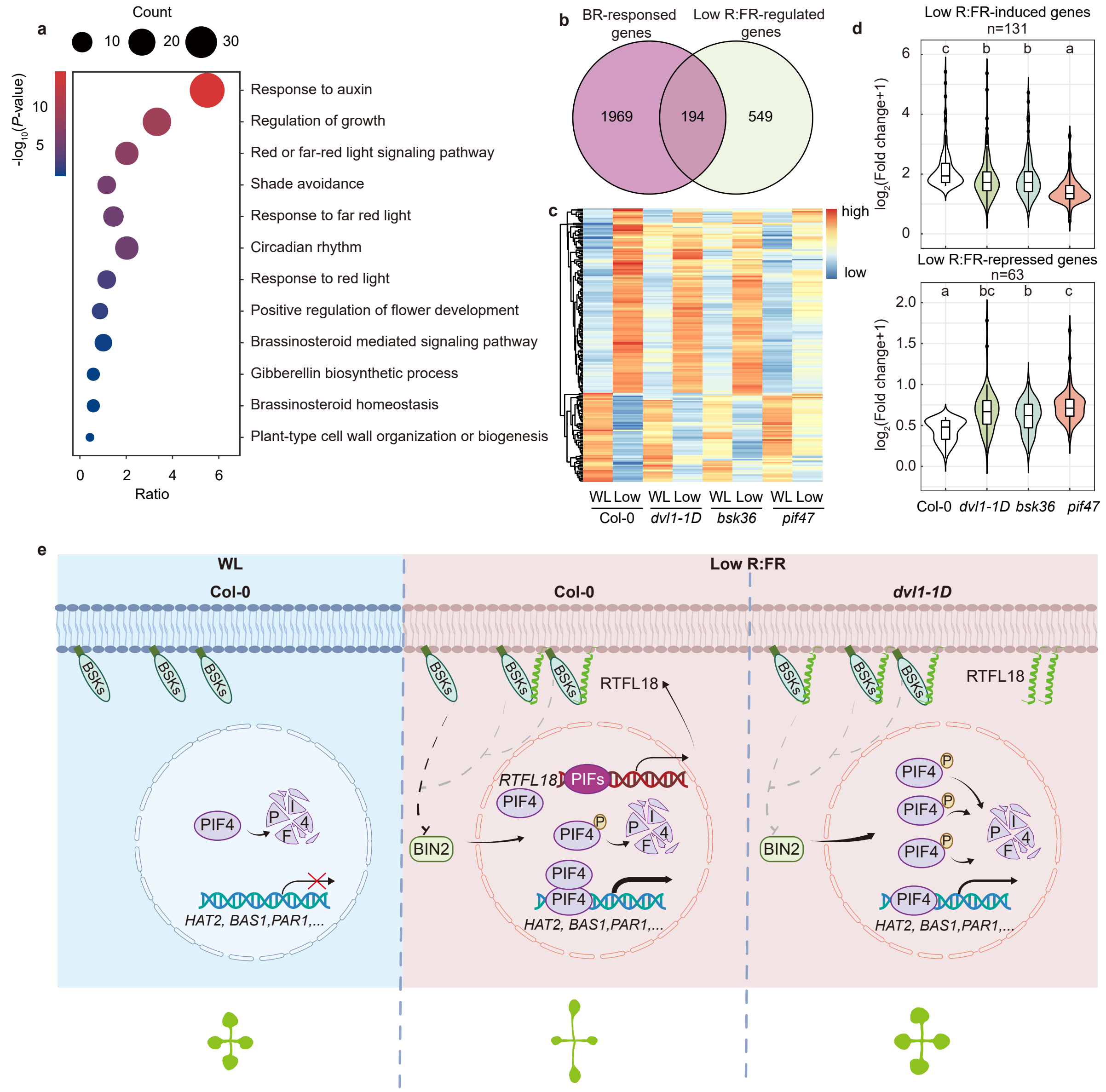

Supplementary Fig. 7 | RTFL18 regulates the expression of BR-responsive genes and a working model of RTFL18. **a**, Gene Ontology (GO) analysis of 743 low R:FR-regulated genes. The size of each point represents the number of genes, and the color represents the *P* value, Fisher's one-tailed test ($P < 0.05$). **b**, Venn diagram representing 194 overlapping genes between low R:FR-regulated genes and BR-responsive genes. **c**, Heatmap showing the transcript levels of 194 overlapping genes in Col-0, *dvl1-1D*, *bsk36*, and *pif47* under white light (WL) and low R:FR conditions. Red, yellow and blue rows indicate RNA expression at high, medium and low levels, respectively. **d**, Boxplot representing the fold changes of 194 BR-responsive genes in Col-0, *dvl1-1D*, *bsk36*, and *pif47*, which were determined by comparing the transcript levels between WL and low R:FR conditions. Boxplots show the median (horizontal line), second to third quartiles (box), and whiskers extend to a maximum of 1.5× interquartile range beyond the box. Letters indicate significant differences between mean values (one-way ANOVA: Tukey's multiple comparisons test, $P < 0.01$), and groups with the same letters are not significantly different. **e**, Proposed working model for the RTFL18-BSK3-PIF4 pathway. Under low R:FR conditions, PIF4 accumulates and regulates the expression of low R:FR-responsive genes, including in *RTFL18*. The polypeptide RTFL18 can interact with BSKs on the cell membrane to negatively regulate BR signaling and prevent an excessive shade avoidance response. In *dvl1-1D*, large amounts of RTFL18 further promote the degradation of PIF4 and suppress low R:FR-induced elongation. Source data are provided as a Source Data file.

References

1. Guo P, Yoshimura A, Ishikawa N, Yamaguchi T, Guo Y, Tsukaya H. Comparative analysis of the RTFL peptide family on the control of plant organogenesis. *J. Plant Res*. 128, 497-510 (2015).

Supplementary Table 1. Primers used in this study.		
	note	Primer sequence
qRT-PCR	*RTFL13*-qPCR-F	ATGAAGATGTCGGAGAGACGAGTTG
	RTFL13-qPCR-R	TCAATAGTCATCCCACCGTAACAAG
	RTFL15-qPCR-F	ATGAAGACGACCGGTTCGAGTGT
	RTFL15-qPCR-R	TTAGTCATGCCAACAAAGAAGCAT
	RTFL16-qPCR-F	ATGGGAGTTTTGAAGAGAAGAGTAT
	RTFL16-qPCR-R	TCAATCTTGCCAACATAGGAGCAT
	RTFL17-qPCR-F	ATGAAGATGGGAGGTTCAAAGAG
	RTFL17-qPCR-R	TCAATCGTGCCAACAAAGAAGCATG
	RTFL18-qPCR-F	ATGGAAATGAAGAGGGTCATGATG
	RTFL18-qPCR-R	TCAATCATGCGAACAAAGGAGCATG
	RTFL19-qPCR-F	ATGGAAAGCATCATGAGCTTGAAGA
	RTFL19-qPCR-R	TTAATCATGCGAACAAAGGAGCATC
	RTFL21-qPCR-F	ATGAAAGGTACCAAGAAGAAGACGC
	RTFL21-qPCR-R	TTAGTCATGCCAACAAATGAGCATG
	BSK3-qPCR-F	GGAGGAATGATCTCTCCAACAGTGTGTG
	BSK3-qPCR-R	CTTCTCCATTCCCAGGATACCAAGGG
	BSK6-qPCR-F	GGAACAATGGTGTCACCAACAGTACACG
	BSK6-qPCR-R	GCATCGGCTTCCATACCCAGCTTG
	PAR1-qPCR-F	GTTTCGAGCGCAGAACCAAAC
	PAR1-qPCR-R	TGCTTCTTCTCGGTCTTCACGTA
	HAT2-qPCR-F	ATGAAGAAGAAGACGGGGGCGAAA
	HAT2-qPCR-R	ACACTTCCACTTGTCTTGCCGTCA
	BAS1-qPCR-F	AATCCAGCTCGGTTTGCGGATG
	BAS1-qPCR-R	AGGCCAAACGGTATGAAGCCAAC
	AT2G39960-F	CCATCGACAGTGCTGATCCA
	AT2G39960-R	CCATTGGGTGACACTTTTGGT
ChIP-qPCR	RTFL18-P1-F	ATCAAGGAGACAAGATACACAAGGT
	RTFL18-P1-R	TCATGTTCCCTCGTTTTCCAATCC
	RTFL18-P2-F	CTACCAAATTAAACAATTCTGCTTT
	RTFL18-P2-R	CTTCATATACTTCCCCAATCTTCTA
	RTFL21-P1-F	ATCCCATTAAGATTACTTTCACCCTTTG
	RTFL21-P1-R	GACATAAAAAAACCCCAGTGATATGAT
	RTFL21-P2-F	CAACATCTCTATGTGCTAAGGCATC
	RTFL21-P2-R	CTTTTGCTCTTTCAAGTATCCTCC
Genotyping	Dvl-LP2	GATGCTTCATAAAACAGAAACTAATC
	Dvl-RP	TTTCAAGCTTTCAAATAACCCGT
	pSKI015-1F	ACCTCCTCGGATTCCATTGCCCAGCT
	rtfl18-id-F	CCTCAAGCATTGTTATATATCAAACGGG
	rtfl18-id-R	GGTTGAGGCCATTATATATACAATTCGG
	rtfl21-id-F	CTCTCTCAATTTCTCTCTGCCACTC
	rtfl21-id-R	ACATGATATGAATGGTTGGAAGCCC
	bsk3-1-LP	CTCACTCCGTAGCTGACCAAC
	bsk3-1-RP	ACGTTCATGTCGATTCCTTTG
	bsk6-LP	ATTGTATCAGAACACGGCGAG
	bsk6-RP	TTCCAACAACTATCCAGCAGG
	LBb1.3	ATTTTGCCGATTTCGGAAC
	Flag-RTFL18-F	ggggatcctctagagtcgacATGGACTACAAGGACGACGATGACAAGCATGAGGATTACAAAGACGATGACGATAAGCAAGGTGACTACAAAGACGATGATGACAAACAAGGTGAAATGAAGAGGGTCATGATG
	Flag-RTFL18-R	gaacgaaagctcgaactagtTCAATCATGCGAACAAAGGAGC
	RTFL17-Flag-F	GGCGGATCCATGAAGATGGGAGGTTCAAAG
	RTFL17-Flag-R	ACGCGTCGACTCAATCGTGCCAACAAAG
	RTFL18-Flag-F	GGCGGATCCATGGAAATGAAGAGGGTCAT
	RTFL18-Flag-R	ACGCGTCGACTCAATCATGCGAACAAAGG
	BSK3-Flag-F	ggacgagctcggtaccATGGGAGGTCAATGCTCTAGC
	BSK3-Flag-R	tggtcgactctagaggatccCTTCACTCGGGGAACTCCATTC
	BSK6-Flag-F	gacgagctcggtaccATGGGAGCTCGTTGCTCAAAGTTC

For cloning	BSK6-Flag-R	tggtcgactctagaggatccGCGCTTGTTACTCTTCTTAGCTTC
	GST-RTFL18-F	cgcgtggatccccggaattcATGGAAATGAAGAGGGTCAT
	GST-RTFL18-R	atgcggccgctcgagtcgacTCAATCATGCGAACAAAGGAG
	18-cLuc-F	gtcccggggcggtaccATGGAAATGAAGAGGGTCATGA
	18-cLuc-R	cgaaagctctgcaggtcgacTCAATCATGCGAACAAAGGAGC
	GFP-RTFL18-F	gacgagctgtacaagATGGAAATGAAGAGGGTCATGA
	GFP-RTFL18-R	cctcttcatttccatCTTGTACAGCTCGTCCATGCCG
	Pro18-F	gggcgaattgggtaccGTGAAACTAATCGCAACGGTGA
	Pro18-R	gctctagaactagtggatccTTTCAAGCTTTCAAATAACCCG
	pSUMO-His-18-F5	agattggtggatccgaattcCACCACCACCACCACCACATGG
	pSUMO-His-18-R5	tggtggtggtggtgctcgagTCAATCATGCGAACAAAGGAGC
	BSK3-GST-F	cgcgtggatccccggaattcATGGGAGGTCAATGCTCTAGC
	BSK3-GST-R	atgcggccgctcgagtcgacTTACTTCACTCGGGGAACTCC
	BSK6-GST-F	cgcgtggatccccggaattcATGGGAGCTCGTTGCTCAAA
	BSK6-GST-R	atgcggccgctcgagtcgacTCAGCGCTTGTTACTCTTCT
	BSK3-cYFP-F	gtacccggggatcctctagaATGGGAGGTCAATGCTCTAGCC
	BSK3-cYFP-R	acgctgccaccgccgtcgacCTTCACTCGGGGAACTCCATTC
	BSK6-cYFP-F	gtacccggggatcctctagaATGGGAGCTCGTTGCTCAAAGT
	BSK6-cYFP-R	acgctgccaccgccgtcgacGCGCTTGTTACTCTTCTTAGCT
	nYFP-RTFL18-F	acgccggcggatcctctagaGAAATGAAGAGGGTCATGATGA
	nYFP-RTFL18-R	cgaaagctctgcaggtcgacTCAATCATGCGAACAAAGGAGC
	BSK3-P2302-F	agctcggtacccggggatccATGGGAGGTCAATGCTCTAGC
	BSK3-P2302-R	ctcaccatactagtgtcgacCTTCACTCGGGGAACTCCATT
	BSK6-P2302-F	agctcggtacccggggatccATGGGAGCTCGTTGCTCAAAGT
	BSK6-P2302-R	ctcaccatactagtgtcgacGCGCTTGTTACTCTTCTTAGCTT
